# Mapping the landscape of autoimmunity and autoinflammation in inborn errors of immunity: broad distribution with distinct clustering patterns

**DOI:** 10.3389/fimmu.2025.1725282

**Published:** 2025-11-28

**Authors:** Azzeddine Tahiat, Souad Touri, Amina Saad-Djaballah, Saliha Hakem, Faiza Fernini, Samira Aggoune, Hayet Belhadj, Chafa Bendahmane, Linda Mokrane, Tahar Bencharif Madani, Zouleikha Benhacine, Souhila Melzi, Rawda Aboura, Hassen Messaoudi, Houda Boudiaf, Mohamed Samir Ladj, Tahar Khelifi Touhami, Lynda Taibi, Samira Zobiri, Rachid Bouhdjila, Zahir Bouzerar, Ouardia Ibsaine, Leila Kedji, Abdelghani Yagoubi, Reda Belbouab, Rachida Boukari, Leila Smati, Sergio D. Rosenzweig, Dusan Bogunovic, Luigi D. Notarangelo, Kamel Djenouhat

**Affiliations:** 1 Department of Medical Biology, Rouiba Hospital, Algiers University of Health Sciences, Algiers, Algeria; 2 Department of Pediatrics, Blida University Hospital, University of Blida, Blida, Algeria; 3 Department of Pediatrics, Bologhine Hospital, Algiers University of Health Sciences, Algiers, Algeria; 4 Department of Pediatrics, Mustapha University Hospital, Algiers University of Health Sciences, Algiers, Algeria; 5 Department of Pediatrics, El-Harrach Hospital, Algiers University of Health Sciences, Algiers, Algeria; 6 Department of Pediatrics, Army Mother and Child Hospital, University of Health Sciences, Algiers, Algeria; 7 Department of Pediatrics, Ain Taya Hospital, Algiers University of Health Sciences, Algiers, Algeria; 8 Department of Pediatrics, Mansourah Hospital, University of Constantine, Constantine, Algeria; 9 Department of Pediatrics, Constantine University Hospital, University of Constantine, Constantine, Algeria; 10 Department of Pediatrics, Bab El-Oued University Hospital, Algiers University of Health Sciences, Algiers, Algeria; 11 Department of Internal Medicine, Rouiba Hospital, Algiers University of Health Sciences, Algiers, Algeria; 12 Department of Pediatric Oncology, Mustapha University Hospital, Algiers University of Health Sciences, Algiers, Algeria; 13 Department of Pediatrics, Birtraria Hospital El Biar, Algiers University of Health Sciences, Algiers, Algeria; 14 Private Practitioner, Constantine, Algeria; 15 Department of Dermatology, Mustapha University Hospital, Algiers University of Health Sciences, Algiers, Algeria; 16 Pediatric Gastroenterology, Centre Algérois de Pédiatrie, Algiers, Algeria; 17 Immunology Service, Department of Laboratory Medicine, Clinical Center, National Institutes of Health, Bethesda, MD, United States; 18 Department of Pediatrics, Center for Genetic Errors of Immunity, Columbia University, New York, NY, United States; 19 National Institute of Allergy and Infectious Diseases, National Institutes of Health, Bethesda, MD, United States

**Keywords:** inborn errors of immunity, autoimmunity, autoinflammation, clustering patterns, autoimmune cytopenia, inflammatory bowel disease, RAG deficiency, CD55 deficiency

## Abstract

**Objective:**

In this study, we analyzed a large cohort of Algerian patients with inborn errors of immunity (IEI) to delineate the burden, spectrum, and distribution of autoimmune and autoinflammatory manifestations.

**Methods:**

This retrospective cohort study was based on recorded data from 825 Algerian patients with IEI. For each patient, autoimmune and autoinflammatory complications occurring before and/or after IEI diagnosis were systematically assessed and documented.

**Results:**

Autoimmune and/or autoinflammatory manifestations were observed in 217 patients (26.3%) and, notably, represented the initial clinical presentation in nearly half. Autoimmune features were documented in 163 patients (19.8%), including 26 (3.2%) with concurrent autoinflammatory findings, whereas isolated autoinflammatory conditions were observed in 54 patients (6.5%). A broad spectrum was observed, with autoimmune cytopenias predominating (11.4%), followed by gastrointestinal (7.8%), rheumatologic (5.3%), and endocrine (3.4%) disorders. Immune dysregulation was a recurrent theme across all IEI categories, with a distinct, disease-specific, clustering of autoimmunity and autoinflammation. Autoimmune cytopenias predominated in T-cell defects, including hypomorphic RAG and CD3γ deficiencies; Inflammatory bowel disease (IBD) was enriched in ARPC1B, DOCK8, and CD55 deficiencies, as well as in chronic granulomatous disease (CGD); endocrine autoimmunity, while a cardinal feature of APECED and IPEX, also characterized STAT1 gain-of-function; inflammatory granulomatous lung disease was a consistent feature in LRBA deficiency; and granulomatous inflammation, whether confined to the lungs or extending to other organs, was prominent in common variable immunodeficiency.

**Conclusion:**

Recognition of clustering patterns, particularly autoimmune cytopenias, IBD, and endocrine autoimmunity, has direct clinical implications. These manifestations should be regarded as red flags, guiding targeted evaluation and genetic testing. Mapping such associations not only refines our understanding of pathogenesis but also provides a practical framework for earlier diagnosis and tailored management.

## 1 Introduction

Inborn errors of immunity (IEI) represent a rapidly expanding group of monogenic disorders that impair the development, function, or regulation of the immune system (1). To date, 576 distinct entities have been described and classified into 10 categories according to their clinical and immunological phenotypes by the International Union of Immunological Societies (IUIS) (2). Historically, these conditions were referred to as primary immunodeficiencies (PIDs) and were primarily defined by recurrent, severe, or unusual infections. Over the past two decades, however, advances in next-generation sequencing (NGS) have transformed our understanding of IEI, revealing an ever-expanding spectrum of disease in which immune dysregulation, manifesting as autoimmunity, autoinflammation, allergy, or malignancy, often predominates over infectious susceptibility (3–5). Recognizing that the term ‘PID’ no longer adequately captures this diversity, the IUIS formally adopted the designation *inborn errors of immunity* in its 2017 classification (6). The current conceptual framework therefore embraces not only susceptibility to infection but also immune dysregulation as integral and coequal dimensions of IEI, reflecting a more encompassing understanding of their pathophysiology (1, 7).

Within the spectrum of IEI, primary immune regulatory disorders (PIRDs) have emerged as a rapidly expanding group in which autoimmunity, often involving multiple organs, alongside recurrent or aberrant inflammation and lymphoproliferation represent the predominant clinical manifestations (8). PIRDs now account for nearly one-quarter of all IEI and include entities characterized by multi-organ autoimmunity, early-onset colitis, autoinflammatory syndromes, and type I interferonopathies, among others (8, 9). Importantly, features of immune dysregulation are also frequently observed in other IEI that were traditionally viewed primarily through the lens of infectious susceptibility, including hypomorphic Recombination Activating Genes (RAG) defects, common variable immunodeficiency (CVID), and activated PI3Kδ syndrome (APDS) (10, 11). These observations underscore that immune dysregulation extends far beyond PIRDs and constitutes a broader, common characteristic of IEI. In line with these observations, a landmark study by Fischer et al., based on data from the French National PIDs Registry reported that 26.2% of 2,183 patients with IEI developed at least one autoimmune or inflammatory complication (12). The risk was strikingly elevated for certain conditions, with a 120-fold increase for autoimmune cytopenias, an 80-fold increase for inflammatory bowel disease (IBD), and a 40-fold increase for arthritis compared with the general population (12).

Autoimmunity and autoinflammation have long been recognized as recurrent themes across IEI. Yet, beyond a handful of registry-based studies that provide predominantly descriptive epidemiological data, the literature remains largely fragmented (12–16). Most available evidence derives from isolated case reports or small series, often limited to selected categories such as T-cell defects, specific subgroups like atypical SCID, or individual entities such as RAG deficiency (17–19). This scattered body of knowledge, while valuable, has not allowed for a unified understanding of the scope and distribution of autoimmunity and inflammation within IEI as a whole. Given these gaps, we aimed through the present study to provide a comprehensive overview of autoimmune and autoinflammatory manifestations in a large, nationally representative cohort of patients with IEI. Our cohort encompasses a wide spectrum of disorders, including T- and B-cell deficiencies as well as innate immunity defects, with a significant representation of complement deficiencies. Importantly, our findings demonstrate that immune dysregulation is not restricted to a single category or subgroup but instead exhibits a heterogeneous distribution, with discernible clustering patterns of autoimmune and autoinflammatory manifestations emerging across the IEI landscape.

## 2 Materials and methods

This retrospective cohort study was conducted on recorded data from 825 Algerian patients with IEI who were diagnosed and registered in the Department of Medical Biology at Rouiba Hospital in Algiers, Algeria, over an eight-year period, from June 2017 to June 2025. The study was approved by the local ethics committee and conducted in accordance with the Declaration of Helsinki. IEI diagnosis was based on the European Society for Immunodeficiencies (ESID) criteria (www.esid.org) and the Middle East and North Africa (MENA) diagnosis guidelines (20). Whenever feasible, genetic testing was performed to identify the underlying genetic defect. Patients were subsequently classified into ten categories according to the 2024 IUIS classification (2).

For each patient, autoimmune and autoinflammatory complications, occurring before and/or after IEI diagnosis, were systematically assessed and recorded. Autoimmune manifestations were defined as clinical or biological features resulting from a breakdown of immune tolerance toward self-antigens, leading to the production of autoantibodies and/or autoreactive lymphocytes and causing tissue- or organ-specific damage. Typical examples included autoimmune cytopenias, endocrinopathies, autoimmune hepatitis, celiac disease, and systemic autoimmune diseases such as systemic lupus erythematosus (SLE). Autoinflammatory manifestations stem from excessive, but not necessarily antigen-specific (i.e., absence of autoantibody production), inflammatory reactions driven by dysregulation of innate immune pathways, including uncontrolled inflammasome activation and cytokine responses. These manifestations encompass IBD or IBD-like conditions, granulomatous inflammation, inflammatory arthritis, inflammatory dermatoses such as psoriasis, pustular psoriasis, and neutrohilic dermatoses, as well as systemic inflammatory diseases, including Behçet disease and IgA vasculitis (Henoch–Schönlein purpura) (21). In some conditions, the distinction between autoimmune and autoinflammatory mechanisms is blurred. For example, Omenn syndrome (OS) displays a mixed pattern, characterized by strong systemic and cutaneous type 2 inflammation alongside autoimmune features driven by central tolerance defects and regulatory T-cell (Treg) dysfunction (22).

Diagnosis of these conditions was based on detailed clinical assessment and complementary paraclinical evaluations, including radiologic examinations, endoscopy, and colonoscopy with biopsy. For autoimmune manifestations, diagnosis additionally relied on autoantibody testing, such as direct Coombs test, detection of platelet-bound antibodies (IgG, IgM, and IgA) by flow cytometry, and antinuclear and anti-tissue antibody testing. All clinical, laboratory, and paraclinical findings were interpreted according to disease-specific, internationally recognized criteria.

Statistical analyses were performed with SPSS software, version 25.0 (IBM, Chicago, IL, USA). Categorical variables were compared using the chi-square test or Fisher’s exact test, as appropriate. Continuous variables were summarized as medians with interquartile ranges (IQRs) and compared using the Kruskal–Wallis test. When overall significance was reached, *post-hoc* pairwise comparisons were performed using Mann–Whitney U tests with Bonferroni correction for multiple comparisons. To illustrate data distribution and clustering patterns, a heatmap was generated in Python using the Seaborn library. Statistical significance was set at a two-sided *P* value <0.05.

## 3 Results

### 3.1 Baseline characteristics

A total of 825 patients with more than 80 distinct IEI were investigated. The cohort comprised 463 males (56.1%) and 362 females (43.9%). The median age at symptom onset was 12 months (IQR, 3–60), with a median age at diagnosis of 68 months (IQR, 19–192). The median age of patients at the last visit was 96 months (IQR, 41.5–217), with a median follow-up period of 18 months (IQR, 11–36). Parental consanguinity was documented in 388 patients (47.0%), reflecting the high prevalence of consanguineous unions in the country and their contribution to the burden of autosomal recessive IEI. Genetic testing was performed in 195 patients (23.6%), revealing pathogenic variants in 155 (79.5%). In the remaining cases, diagnosis relied on characteristic clinical features and immunological investigations according to ESID criteria and MENA diagnosis guidelines. Combined immunodeficiencies (CIDs) were the most frequent (208 patients, 25.2%), followed by predominantly antibody deficiencies (PADs; 198 patients, 24.0%) and complement deficiencies (155 patients, 18.8%). Among individual entities, the most common were hereditary angioedema (HAE; 115 patients, 13.9%), CVID (81 patients, 9.8%), hyper-IgE syndrome (HIES; 74 patients, 9.0%), MHC class II deficiency (43 patients, 5.2%), selective IgA deficiency (30 patients, 3.6%), and T^–^B^–^NK^+^ severe combined immunodeficiency (T^–^B^–^NK^+^ SCID; 24 patients, 2.9%) (**Supplementary Table S1**).

### 3.2 Overview of autoimmune and autoinflammatory manifestations in the cohort

**Figure 1:**
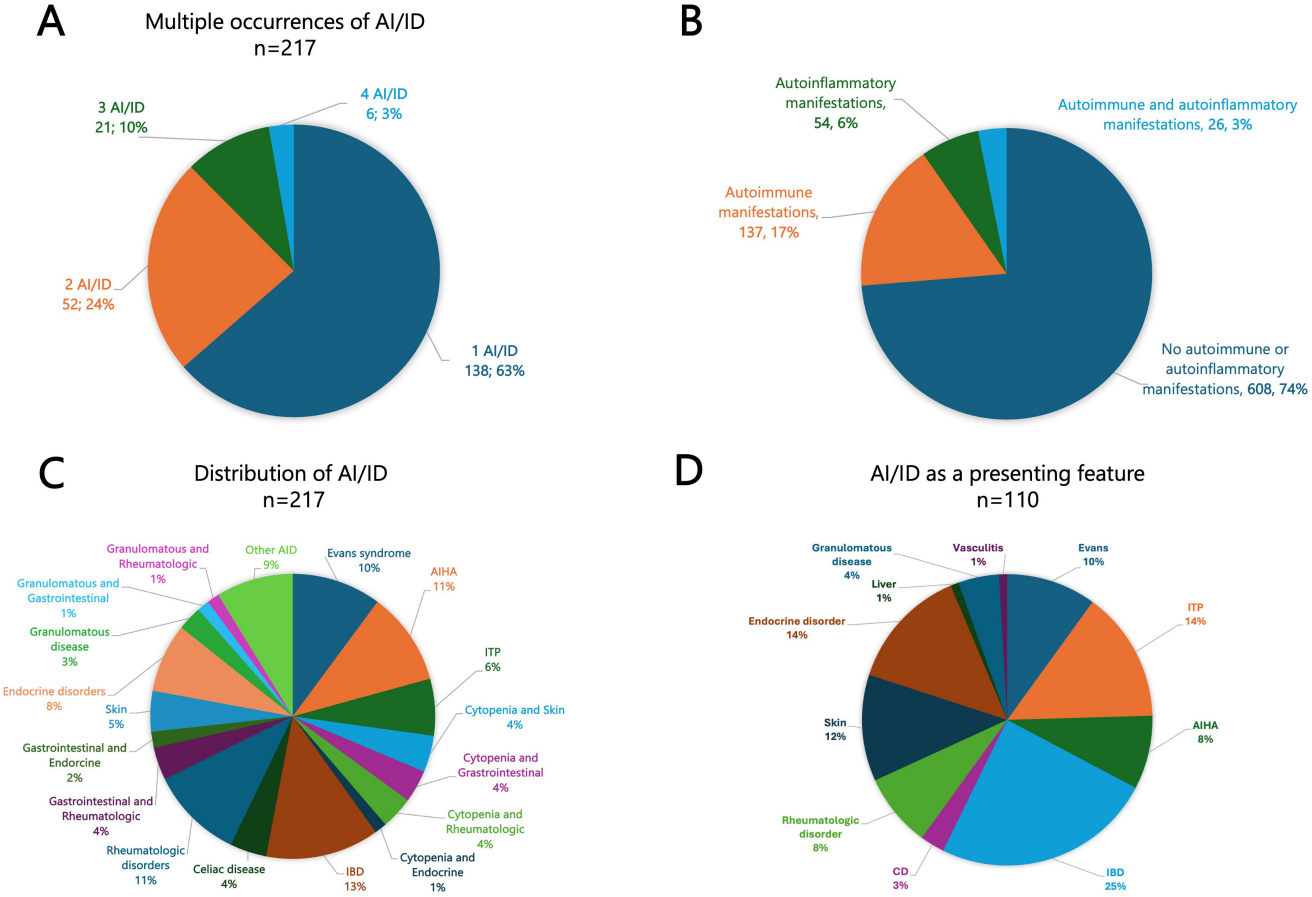
**(A)** Pie chart illustrating the frequency of multiple occurrences of autoimmune and/or autoinflammatory manifestations (ranging from 1 to 4). **(B)** Overall frequency of autoimmune and autoinflammatory manifestations in our cohort. **(C)** Distribution of AI/ID among 217 IEI patients presenting with autoimmune and/or autoinflammatory features. **(D)** Frequency and distribution of autoimmune and autoinflammatory conditions among 110 patients with autoimmunity and/or autoinflammation as the presenting feature. *AI/ID, Autoimmune/autoinflammatory diseases; AIHA, Autoimmune hemolytic anemia; CD, Celiac disease; IBD, Inflammatory bowel disease; ITP, Immune thrombocytopenia*.

Of the 825 patients, 217 (26.3%) experienced at least one autoimmune or autoinflammatory complication, including 138 (63.6%) with a single manifestation, 52 (24.0%) with two, 21 (9.7%) with three, and 6 (2.8%) with four. Autoimmune manifestations were observed in 163 patients (19.8%), including 26 (3.2%) with overlapping autoinflammatory features, whereas isolated autoinflammatory conditions occurred in 54 patients (6.5%) (**Figures 1A, B**). The spectrum was remarkably broad, encompassing more than 30 distinct autoimmune and autoinflammatory entities. Autoimmune cytopenias (11.4%), gastrointestinal disorders (7.8%), rheumatologic diseases (5.3%), and autoimmune endocrinopathies (3.4%) accounted for the most frequent immune dysregulation features (**Table 1**, **Figure 1C**). Among autoimmune conditions, autoimmune hemolytic anemia (AIHA, 5.5%), Evans syndrome (3.4%), and immune thrombocytopenia (ITP, 2.4%) predominated, followed by celiac disease (2.4%), Hashimoto’s thyroiditis (1.6%), type 1 diabetes (1.5%), and SLE (1.5%). Autoinflammatory manifestations were primarily represented by IBD (5.2%), granulomatous inflammation (2.1%), and inflammatory arthritis (1.5%) (**Table 1**).

**Table 1:** Overall frequencies of autoimmune and autoinflammatory conditions in our cohort.

Immune dysregulation feature	Autoimmune/ autoinflammatory	No.	Percent
Autoimmune cytopenia		94	11.4
Autoimmune hemolytic anemia	Autoimmune	45	5.5
Evans syndrome	Autoimmune	28	3.4
Immune thrombocytopenia	Autoimmune	20	2.4
Autoimmune neutropenia	Autoimmune	1	0.1
Gastrointestinal disorders		64	7.8
Inflammatory bowel disease	Autoinflammatory	43	5.2
Celiac disease	Autoimmune	20	2.4
Autoimmune enteropathy	Autoimmune	1	0.1
Rheumatologic disorders		44	5.3
Systemic lupus erythematosus	Autoimmune	12	1.5
Juvenile idiopathic arthritis	Autoimmune	9	1.1
Rheumatoid arthritis	Autoimmune	4	0.5
Systemic sclerosis	Autoimmune	3	0.4
Sjögren’s syndrome	Autoimmune	2	0.2
Undifferentiated connective tissue disease	Autoimmune	2	0.2
Inflammatory arthritis	Autoinflammatory	12	1.5
Endocrine disorders		28	3.4
Hashimoto's thyroiditis	Autoimmune	13	1.6
Type 1 diabetes	Autoimmune	12	1.5
Autoimmune polyendocrine syndrome type 1	Autoimmune	2	0.2
Graves’ disease	Autoimmune	1	0.1
Addison’s disease	Autoimmune	1	0.1
Skin		22	2.7
OS–associated erythroderma	Autoinflammatory	8	1.0
Psoriasis	Autoinflammatory	5	0.6
Neutrophilic dermatoses	Autoinflammatory	3	0.4
Bullous pemphigoid	Autoimmune	2	0.2
Pemphigus	Autoimmune	2	0.2
Vitiligo-like depigmentation	Autoimmune	1	0.1
Pustular psoriasis	Autoinflammatory	1	0.1
Granulomatous disease	Autoinflammatory	17	2.1
Lung		15	1.8
Inflammatory granulomatous lung disease	Autoinflammatory	15	1.8
Liver		10	1.2
Autoimmune hepatitis	Autoimmune	10	1.2
Neurologic disorders		3	0.4
Autoimmune encephalitis	Autoimmune	1	0.1
CNS demyelinating disease	Autoimmune	1	0.1
Immune-mediated peripheral neuropathy	Autoimmune	1	0.1

CNS, Central nervous system; OS, Omenn syndrome.

The median age at onset of the first autoimmune or autoinflammatory manifestation was 60 months (IQR, 12–168), with significant variation across IEI categories (*P* < 0.0001). *Post-hoc* pairwise analyses demonstrated that patients with PADs developed their first autoimmune or autoinflammatory manifestation later than those with CID, CID with syndromic features, and diseases of immune dysregulation. Patients with complement deficiencies exhibited an even more delayed onset compared with CID, CID with syndromic features, diseases of immune dysregulation, and defects of intrinsic and innate immunity (All significant pairwise comparisons had raw *P*-values <0.0001; **Supplementary Table S2**).

Autoimmune and/or autoinflammatory manifestations preceded the recognition of IEI in 58.5% of cases, coincided with the diagnosis in 11.1%, and constituted the very first clinical manifestation in 50.7% of affected patients, accounting for 13.3% of the entire cohort. The most common inaugural presentations included IBD (24.5%), ITP (14.5%), Evans syndrome (10.0%) and AIHA (8.2%) (**Figure 1D**).

### 3.3 Broad distribution of autoimmune and autoinflammatory manifestations across IEI

**Figure 2:**
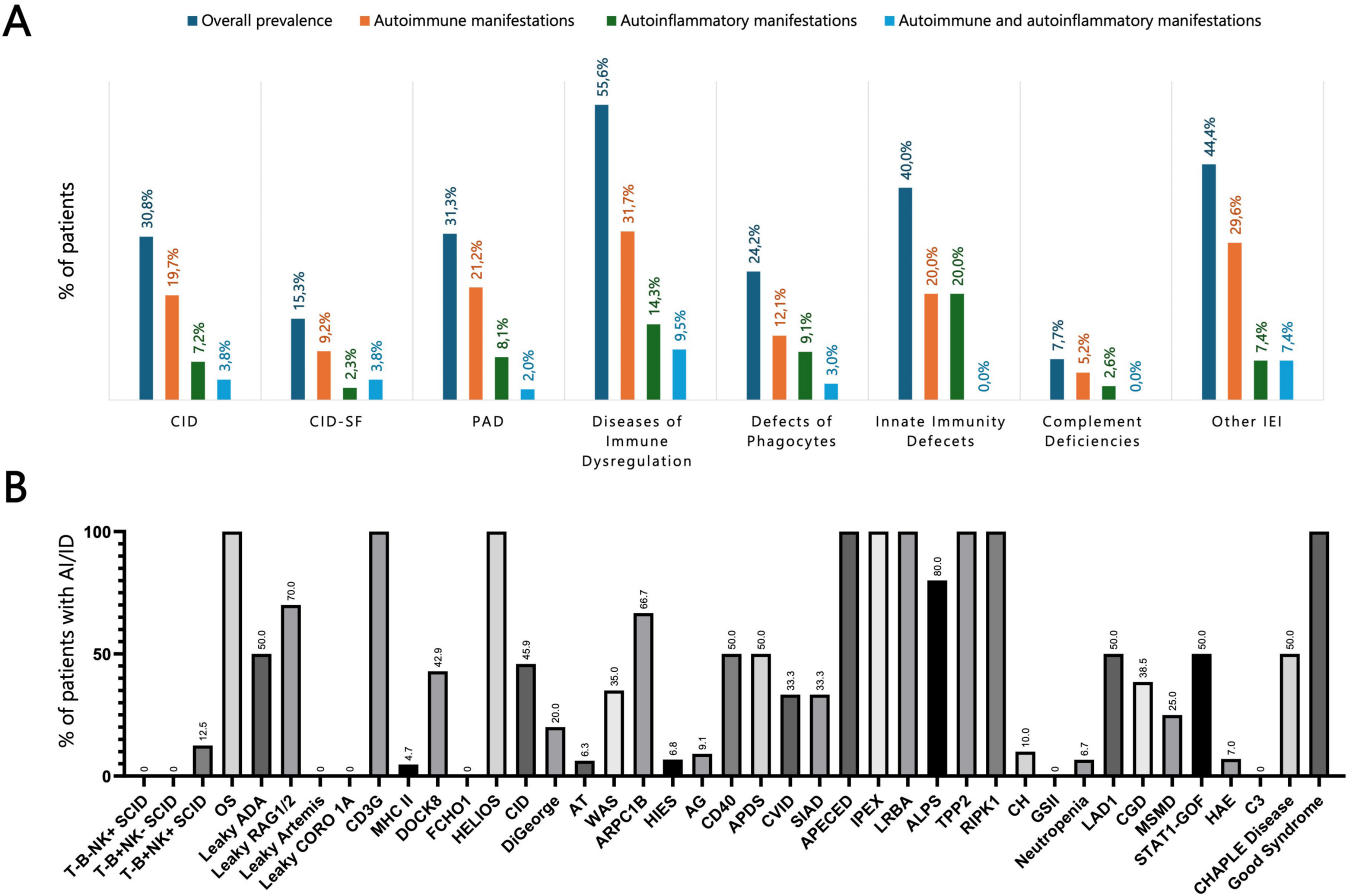
**(A)** Prevalence of autoimmune and autoinflammatory manifestations across different IEI categories. **(B)** Prevalence of autoimmune and autoinflammatory complications in a selected panel of IEI, representing the most common entities in our cohort. *ADA, Adenosine deaminase; AG, Agammaglobulinemia; AI/ID, Autoimmune/autoinflammatory diseases; ALPS, Autoimmune lymphoproliferative syndrome; APDS, Activated PI3K delta syndrome; APECED, Autoimmune polyendocrinopathy–candidiasis–ectodermal dystrophy; ARPC1B, Actin related protein 2/3 complex subunit 1B; AT, Ataxia-telangiectasia; CGD, Chronic granulomatous disease; CH, Chediak-Higashi; CHAPLE, Complement hyperactivation angiopathic thrombosis and protein-losing enteropathy; CID, Combined immunodeficiency; CID-SF, Combined immunodeficiency with associated or syndromic features; CORO1A, Coronin 1A; CVID, Common variable immunodeficiency; DOCK8, Dedicator of cytokinesis 8; FCHO1, F-BAR domain only protein 1; GSII, Griscelli syndrome type II; HAE, Hereditary angioedema; HIES, Hyper-IgE syndrome; IEI, Inborn errors of immunity; IPEX, Immune dysregulation–polyendocrinopathy–enteropathy–X-linked; LAD, Leukocyte adhesion deficiency; LRBA, LPS-responsive beige-like anchor protein; MHC, Major histocompatibility complex; MSMD, Mendelian susceptibility to mycobacterial disease; OS, Omenn syndrome; PAD, Predominantly antibody deficiency; RAG, Recombination activating gene; RIPK1, Receptor-interacting serine/threonine-protein kinase 1; SCID, Severe combined immunodeficiency; SIAD, Selected IgA deficiency; STAT, Signal transducer and activator of transcription; TPP2, Tripeptidyl peptidase II; WAS, Wiskott-Aldrich syndrome*.

The analysis of autoimmune and autoinflammatory manifestations in our cohort revealed that these complications were not confined to a single IEI category but extended across the entire IEI spectrum. They were particularly frequent in PIRDs but were also prominent among patients with CID and PAD. In CIDs, such manifestations were observed in 30.8% of cases, including 19.7% with autoimmune manifestations, 7.2% with autoinflammatory features, and 3.8% with both concurrently. Within the PAD group, these manifestations were comparably prevalent, affecting 31.3% of patients, with 21.2% exhibiting autoimmune features, 8.1% autoinflammatory manifestations, and 2.0% both. Interestingly, autoimmune (12.1%) and autoinflammatory (9.1%) manifestations were also identified in nearly one quarter of patients with congenital defects of phagocytes (24.2%) and were present, though less frequently, in complement deficiencies (7.7%) (**Figures 2A, B**).

#### 3.3.1 Diseases of immune dysregulation

This heterogeneous group encompasses syndromes of early-onset polyautoimmunity, such as immune dysregulation, polyendocrinopathy, enteropathy, X-linked (IPEX) syndrome and LPS-Responsive Beige-Like Anchor protein (LRBA) deficiency, as well as conditions characterized by other forms of immune dysregulation, including chronic lymphoproliferation (e.g., X-linked lymphoproliferative syndrome) and hemophagocytic lymphohistiocytosis (HLH), encompassing familial HLH and Chediak–Higashi syndrome (2). Among the 63 patients in this group, autoimmune manifestations occurred in 20 (31.7%), autoinflammatory conditions in 9 (14.3%), and both in 6 (9.5%), for an overall prevalence of 55.6%. The most frequent findings were autoimmune cytopenias (30.2%), gastrointestinal involvement (23.8%), and endocrine disorders (14.3%) (**Supplementary Table S3**).

#### 3.3.2 Combined immunodeficiency syndromes

**Figure 3:**
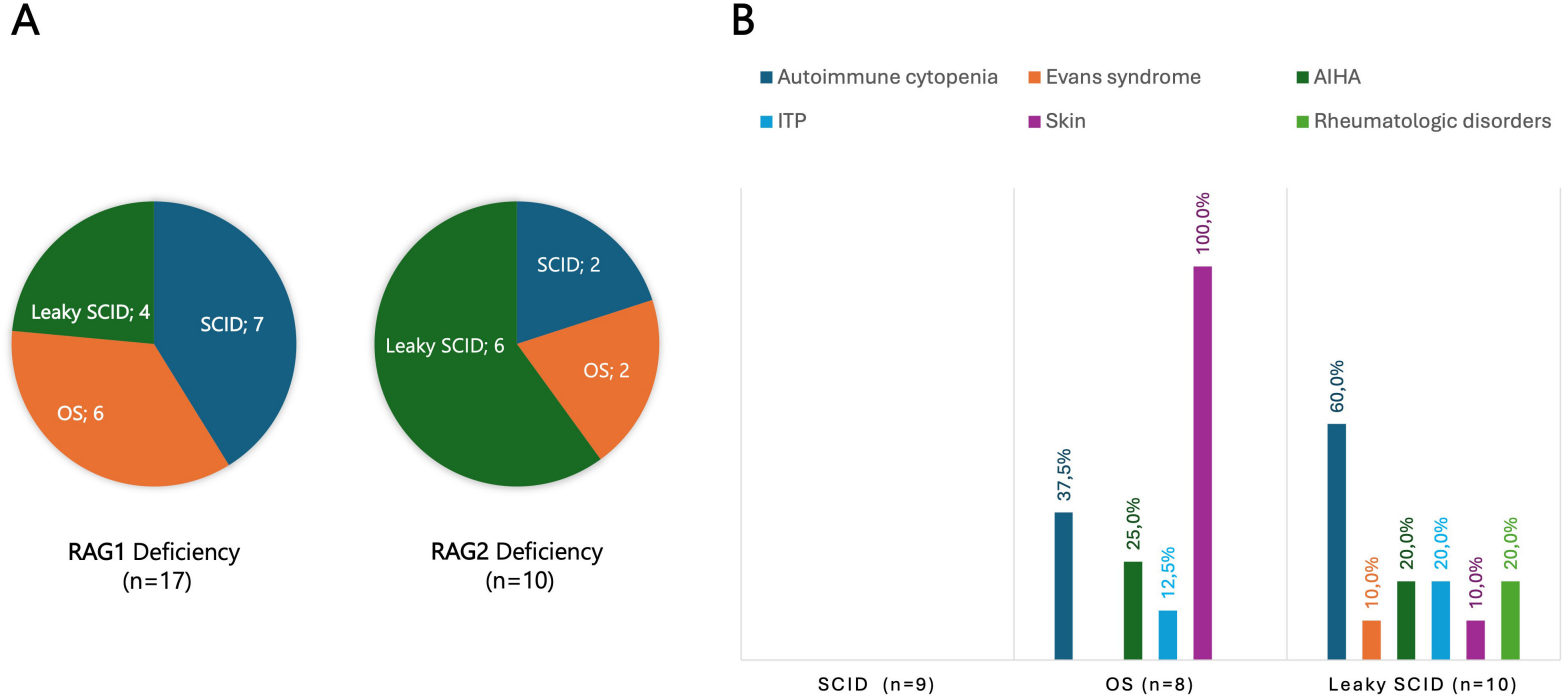
**(A)** Disease forms in 17 patients with RAG1 and 10 patients with RAG2 deficiencies. **(B)** Autoimmune/autoinflammatory manifestations in classical and atypical forms of SCID, including Omenn syndrome and leaky SCID. Skin involvement was a constant (100%) feature in Omenn syndrome, whereas autoimmune cytopenias were observed in 60% of patients with leaky SCID. *AIHA, Autoimmune hemolytic anemia; ITP, Immune thrombocytopenia; OS, Omenn syndrome; RAG, Recombination activating gene; SCID, Severe combined immunodeficiency*.

Among 208 patients with CID, 64 (30.8%) experienced at least one autoimmune or autoinflammatory complication. Autoimmune cytopenias (16.8%) and cutaneous involvement (7.7%) were most frequent, followed by gastrointestinal (4.8%) and rheumatologic manifestations (3.8%) (**Supplementary Table S3**). Features of immune dysregulation emerged as a recurrent theme across the spectrum of T-cell immunodeficiencies, though their prevalence varied markedly between subtypes. Autoimmune manifestations were uncommon in classical SCID (4.9%) but occurred more frequently in atypical forms, reflecting a greater propensity for immune dysregulation. Among patients with leaky SCID, 8 of 19 (42.1%) developed autoimmune features. The highest risk was observed in those carrying hypomorphic RAG1 or RAG2 variants (70%), most commonly presenting with autoimmune cytopenias (60%) (**Figures 3A, B**). Among *bona fide* CIDs, autoimmune and/or autoinflammatory manifestations were observed in 32.9% of cases. Their distribution varied substantially across genetic entities: CD3γ, Dedicator of Cytokinesis 8 (DOCK8), and Helios deficiencies were consistently associated with high frequencies of autoimmunity, whereas other causes, such as MHC class II and F-BAR domain-only protein 1 (FCHO1) deficiencies, were only rarely linked to immune dysregulation (**Table 2**).

**Table 2:** Distribution of autoimmune and autoinflammatory manifestations across IEI types.

IEI	No.	Overall (AI/ID) (%)	Autoimmune diseases (%)	Autoinflammatory diseases (%)	Autoimmune cytopenia (%)	Gastrointestinal disorders (%)	Skin (%)	Rheumatologic disorders (%)	Endocrine disorders (%)	Granulomatous disease (%)
Combined immunodeficiency syndromes										
SCIDs	41	4.9	4.9	0.0	4.9	0.0	0.0	0.0	0.0	0.0
T^–^B^–^NK^+^ SCID	24	0.0	0.0	0.0	0.0	0.0	0.0	0.0	0.0	0.0
T^–^B^+^NK^–^ SCID	7	0.0	0.0	0.0	0.0	0.0	0.0	0.0	0.0	0.0
T^–^B^+^NK^+^ SCID	8	12.5	12.5	0.0	12.5	0.0	0.0	0.0	0.0	0.0
Omenn syndrome	8	100	37.5	100	37.5	0.0	100	0.0	0.0	0.0
Leaky SCIDs	19	42.1	42.1	0.0	36.8	0.0	10.5	10.5	0.0	0.0
Hypomorphic RAG deficiency	10	70.0	70.0	0.0	60.0	0.0	10.0	20.0	0.0	0.0
Hypomorphic Artemis deficiency	3	0.0	0.0	0.0	0.0	0.0	0.0	0.0	0.0	0.0
Hypomorphic ADA deficiency	2	50.0	50.0	0.0	50.0	0.0	50.0	0.0	0.0	0.0
Hypomorphic Coronin 1A deficiency	2	0.0	0.0	0.0	0.0	0.0	0.0	0.0	0.0	0.0
*Bona fide* CIDs	140	32.9	25.7	10.7	16.4	7.1	4.3	4.3	1.4	2.1
MHC class II deficiency	43	4.7	4.7	0.0	2.3	2.3	0.0	0.0	0.0	0.0
DOCK8 deficiency	7	42.9	28.6	14.3	0.0	28.6	0.0	14.3	0.0	0.0
CD3γ deficiency	2	100	100	0.0	100	0.0	0.0	0.0	0.0	0.0
Helios deficiency	2	100	100	50.0	50.0	50.0	50.0	0.0	0.0	0.0
FCHO1 deficiency	3	0.0	0.0	0.0	0.0	0.0	0.0	0.0	0.0	0.0
CIDs with associated or syndromic features	131	15.3	13.0	6.1	9.9	6.1	0.8	1.5	0.8	0.0
DiGeorge syndrome	10	20.0	20.0	10.0	20.0	0.0	0.0	10.0	0.0	0.0
Ataxia Telangiectasia	16	6.3	6.3	0.0	0.0	0.0	0.0	0.0	0.0	0.0
Wiskott-Aldrich syndrome	20	35.0	30.0	20.0	30.0	20.0	0.0	0.0	0.0	0.0
ARPC1B deficiency	3	66.7	33.3	66.7	33.3	66.7	0.0	0.0	0.0	0.0
Hyper-IgE syndrome	74	6.8	5.1	1.4	2.7	2.7	1.4	1.4	0.0	0.0
Primary antibody deficiency syndromes										
Agammaglobulinemia	22	9.1	4.5	4.5	4.5	0.0	0.0	4.5	0.0	0.0
Activated PI3K delta syndrome	4	50.0	50.0	0.0	25.0	25.0	0.0	0.0	0.0	0.0
CVID	81	33.3	19.8	14.8	11.1	12.3	2.5	3.7	3.7	9.9
Selective IgA deficiency	30	33.3	30.0	3.3	3.3	10.0	0.0	13.3	10.0	0.0
Diseases of immune dysregulation										
APECED	2	100	100	0.0	0.0	0.0	0.0	0.0	100	0.0
IPEX	1	100	100	0.0	100	100	0.0	0.0	100	0.0
ALPS	5	80.0	80.0	0.0	80.0	0.0	0.0	0.0	0.0	0.0
LRBA deficiency	3	100	100	100	100	0.0	0.0	33.3	33.3	100
TPP2 deficiency	2	100	100	0.0	100	0.0	0.0	50.0	50.0	0.0
RIPK1 deficiency	5	100	0.0	100	0.0	100	0.0	0.0	0.0	0.0
Innate immunity defects										
Congenital defects of phagocytes	33	24.2	15.2	12.1	9.1	12.1	0.0	0.0	6.1	0.0
Congenital neutropenia	15	6.7	6.7	0.0	6.7	0.0	0.0	0.0	0.0	0.0
Chronic granulomatous disease	13	38.5	15.4	30.8	0.0	23.1	0.0	0.0	15.4	0.0
Defects in intrinsic and innate immunity	10	40.0	20.0	20.0	0.0	0.0	0.0	10.0	20.0	0.0
MSMD	4	25.0	0.0	25.0	0.0	0.0	0.0	0.0	0.0	0.0
STAT1 GOF	4	50.0	50.0	0.0	0.0	0.0	0.0	0.0	50.0	0.0
Complement deficiencies	155	7.7	5.2	2.6	0.0	5.2	0.0	1.9	1.3	0.0
HAE	115	7.0	6.1	0.9	0.0	4.3	0.0	2.6	0.9	0.0
CD55 deficiency (CHAPLE disease)	8	50.0	12.5	37.5	0.0	37.5	0.0	0.0	12.5	0.0
C3 deficiency	2	0.0	0.0	0.0	0.0	0.0	0.0	0.0	0.0	0.0
C7 deficiency	1	0.0	0.0	0.0	0.0	0.0	0.0	0.0	0.0	0.0

ADA, Adenosine deaminase; AI/ID, Autoimmune/autoinflammatory diseases; ALPS, Autoimmune lymphoproliferative syndrome; APECED, Autoimmune polyendocrinopathy–candidiasis–ectodermal dystrophy; ARPC1B, Actin related protein 2/3 complex subunit 1B; CHAPLE, Complement hyperactivation angiopathic thrombosis and protein-losing enteropathy; CID, Combined immunodeficiency; CVID, Common variable immunodeficiency; DOCK8, Dedicator of cytokinesis 8; FCHO1, F-BAR domain only protein 1; GOF, Gain-of-function; HAE, Hereditary angioedema; IEI, Inborn errors of immunity; IPEX, Immune dysregulation–polyendocrinopathy–enteropathy–X-linked; LRBA, LPS-responsive beige-like anchor protein; MHC, Major histocompatibility complex; MSMD, Mendelian susceptibility to mycobacterial disease; RAG, Recombination activating gene; RIPK1, Receptor-interacting serine/threonine-protein kinase 1; SCID, Severe combined immunodeficiency; STAT, Signal transducer and activator of transcription; TPP2, Tripeptidyl peptidase II.

Among CIDs with associated or syndromic features, autoimmune and autoinflammatory complications were most frequent in actin-related disorders, including Wiskott–Aldrich syndrome (35.0%) and Actin-Related Protein 2/3 Complex Subunit 1B (ARPC1B) deficiency (66.7%), with autoimmune cytopenias and gastrointestinal disease as the predominant complications. In contrast, these manifestations were uncommon in Ataxia–Telangiectasia (6.3%) and HIES (6.8%). An intermediate prevalence was noted in DiGeorge syndrome, with 2 of 10 patients (20%) developing autoimmune cytopenias, including one case revealed by refractory ITP (**Table 2**).

#### 3.3.3 Primary antibody deficiency syndromes

Nearly one-third of patients with PAD exhibited autoimmune and/or autoinflammatory manifestations, most commonly rheumatologic disorders (10.6%), autoimmune cytopenias (10.6%), gastrointestinal disease (8.1%), and granulomatous complications (4.5%). Autoimmune phenomena were rare in agammaglobulinemia but particularly frequent in activated PI3Kδ syndrome, and occurred at relatively high frequencies in CVID (33.3%) and selective IgA deficiency (33.3%). In CVID, gastrointestinal (12.3%) and granulomatous disease (9.9%) predominated, whereas rheumatologic manifestations were most common in selective IgA deficiency (13.3%) (**Table 2**).

#### 3.3.4 Innate immunity defects

Innate immunity defects span three IUIS categories: congenital defects of phagocytes, complement deficiencies, and defects in intrinsic and innate immunity. The latter group comprises disorders marked by narrowly defined patterns of susceptibility to infections, often restricted to specific pathogens (2). In patients with STAT1 gain-of-function mutations, chronic mucocutaneous candidiasis was associated with frequent autoimmune endocrine disease, most often Hashimoto thyroiditis (2 of 4 cases, 50%). Among 15 patients with congenital neutropenia, autoimmunity was rare, documented in only one case (Evans syndrome). By contrast, autoimmune and autoinflammatory complications were noted in 5 of 13 patients with chronic granulomatous disease (CGD) (38.5%), most commonly IBD, which affected 3 patients (23.1%) and constituted the initial presentation in one case (**Table 2**).

HAE, caused by C1 inhibitor deficiency, was by far the most common complement deficiency, accounting for 115 of 155 cases in our series. Although the overall prevalence of autoimmunity in HAE was modest compared with other IEI, celiac disease and SLE were documented in 2.6% and 1.7% of patients, respectively. By contrast, CD55 deficiency-also known as CHAPLE (Complement Hyperactivation, Angiopathic Protein-Losing Enteropathy) disease-was marked by a high prevalence of immune dysregulation features, with IBD-like inflammatory changes documented in three patients (37.5%). Bowel inflammation was evidenced by endoscopic findings of ulcerations and micronodules, along with radiologic signs of stenosing and multifocal ileal inflammation on CT and MR enterography. Histology revealed ileal inflammatory infiltrates, with erosive colitis identified in one patient. In addition, Hashimoto’s disease was observed in one other patient, bringing the total number of patients with immune dysregulation features to four (**Table 2**).

### 3.4 Clustering patterns of autoimmune and autoinflammatory manifestations across the IEI spectrum

**Figure 4:**
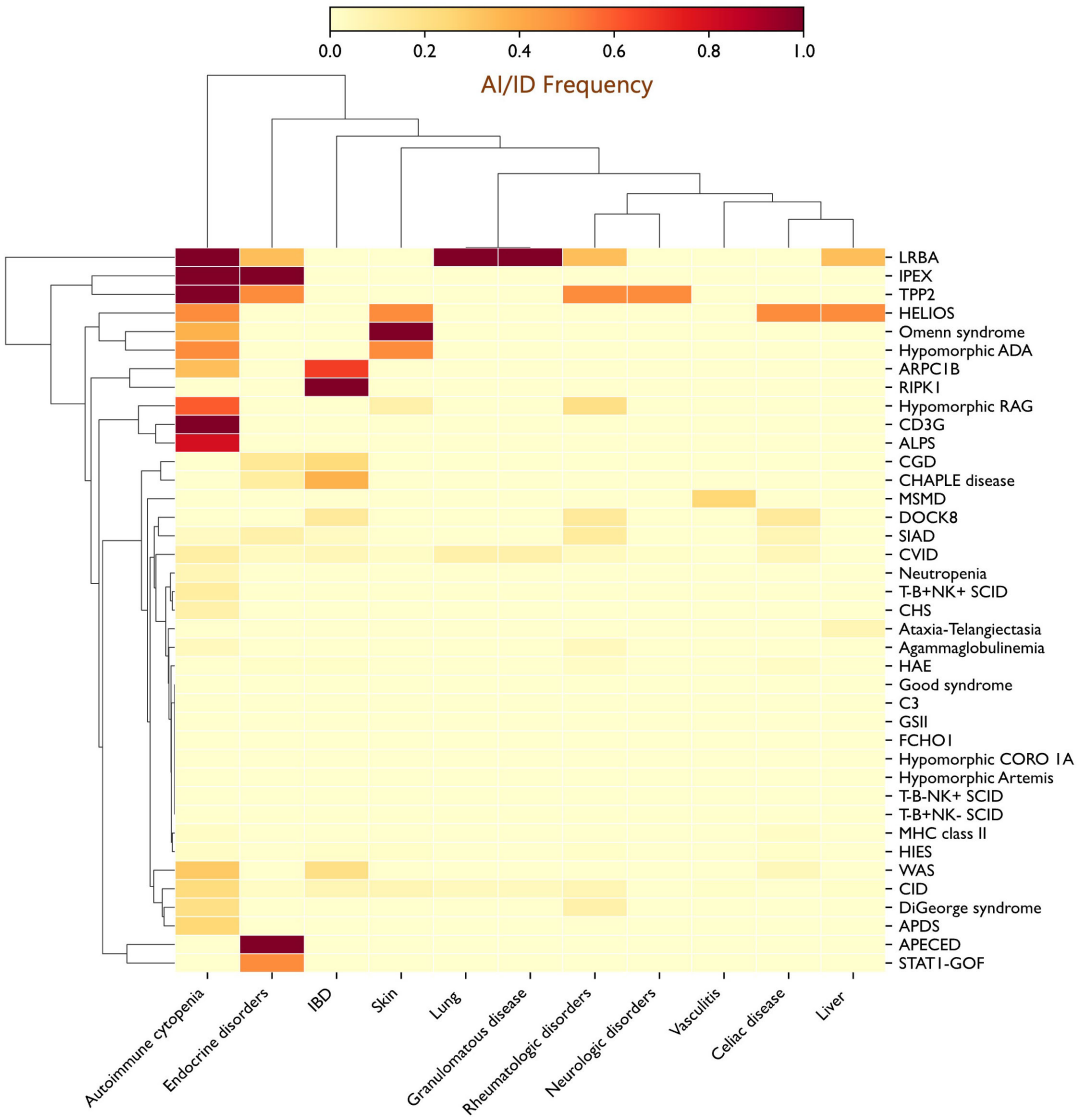
Heatmap depicting the distribution of autoimmune and autoinflammatory manifestations across the IEI spectrum, revealing clustering of specific autoimmune features with distinct genetic defects. Autoimmune cytopenias were enriched in PIRDs such as ALPS, LRBA, and TPP2 deficiencies, as well as in T-cell defects, including CD3γ and RAG deficiencies; IBD was frequent in ARPC1B deficiency, CHAPLE disease, and CGD; and endocrine autoimmunity was prominent in patients with STAT1 gain-of-function (GOF) mutations. Additional notable associations included skin involvement in OS (100%) and inflammatory granulomatous lung disease in LRBA deficiency (100%). *ADA, Adenosine deaminase; ALPS, Autoimmune lymphoproliferative syndrome; APDS, Activated PI3K delta syndrome; APECED, Autoimmune polyendocrinopathy–candidiasis–ectodermal dystrophy; ARPC1B, Actin related protein 2/3 complex subunit 1B; CGD, Chronic granulomatous disease; CHS, Chediak-Higashi syndrome; CHAPLE, Complement hyperactivation angiopathic thrombosis and protein-losing enteropathy; CID, Combined immunodeficiency; CORO1A, Coronin 1A; CVID, Common variable immunodeficiency; DOCK8, Dedicator of cytokinesis 8; FCHO1, F-BAR domain only protein 1; GSII, Griscelli syndrome type II; HAE, Hereditary angioedema; HIES, Hyper-IgE syndrome; IBD, Inflammatory bowel disease; IPEX, Immune dysregulation–polyendocrinopathy–enteropathy–X-linked; LRBA, LPS-responsive beige-like anchor protein; MHC, Major histocompatibility complex; MSMD, Mendelian susceptibility to mycobacterial disease; OS, Omenn syndrome; RAG, Recombination activating gene; RIPK1, Receptor-interacting serine/threonine-protein kinase 1; SCID, Severe combined immunodeficiency; SIAD, Selected IgA deficiency; STAT, Signal transducer and activator of transcription; TPP2, Tripeptidyl peptidase II; WAS, Wiskott-Aldrich syndrome*.

Analysis of the distribution of autoimmune and autoinflammatory manifestations across IEI categories revealed significant intergroup differences, with preferential associations between specific autoimmune/autoinflammatory conditions and distinct IEI categories or particular genetic defects (**Table 3**, **Supplementary Figure S1**). Autoimmune cytopenias were particularly frequent in disorders of immune dysregulation, notably LRBA (100%) and Tripeptidyl peptidase II (TPP2) (100%) deficiencies, and autoimmune lymphoproliferative syndrome (ALPS, 80%), but were also strikingly common in T-cell defects, including CD3γ deficiency (100%) and hypomorphic RAG deficiency (60%). IBD emerged as a hallmark of certain PIRDs, such as Receptor-Interacting Serine/Threonine-Protein Kinase 1 (RIPK1) deficiency (100%), but was also common in immune-related actinopathies, including ARPC1B (66.7%) and dedicator of cytokinesis 8 (DOCK8, 14.3%) deficiencies, as well as in selected innate immunity defects, particularly CGD (23.1%) and CHAPLE disease (37.5%). Endocrine autoimmunity is a defining feature of Autoimmune Polyendocrinopathy-Candidiasis-Ectodermal Dystrophy (APECED) and IPEX syndrome, but was also prominent in patients with STAT1 gain-of-function variants. Granulomatous disease, primarily affecting the lungs and extending in some cases to other organs (e.g., liver and spleen), was more prevalent among PADs, especially CVID (9.9%). Additional notable associations included skin manifestations (100%) in OS and lung involvement (100%), manifesting as granulomatous–lymphocytic interstitial lung disease (GLILD), in LRBA deficiency. These patterns are summarized in the heatmap, providing a visual overview of co-occurrence trends across different genetic defects (**Figure 4**).

**Table 3:** Prevalence and distribution of autoimmune and autoinflammatory conditions with respect to IEI categories.

AI/ID	CIDs	CIDs with syndromic features	PADs	Diseases of immune dysregulation	Congenital defects of phagocytes	Defects in intrinsic and innate immunity	Complement deficiencies	*P*
Autoimmune cytopenia (%)	16.8	9.9	10.6	30.2	9.1	0.0	0.0	<0.0001
Gastrointestinal disease (%)	4.8	6.1	8.1	23.8	12.1	0.0	5.2	<0.0001
IBD (%)	3.4	5.3	4.0	19.0	9.1	0.0	2.6	<0.0001
Skin (%)	7.7	0.8	1.5	1.6	0.0	0.0	0.0	<0.0001
Rheumatological disease (%)	3.8	1.5	10.6	9.5	0.0	10.0	1.9	<0.0001
Endocrine disease (%)	1.0	0.8	4.5	14.3	6.1	20.0	1.3	<0.0001
Granulomatous disease (%)	1.4	0.0	4.5	6.3	0.0	0.0	0.0	0.003
Lung (%)	1.4	0.0	4.5	4.8	0.0	0.0	0.0	0.009
Liver (%)	1.4	0.8	0.0	6.3	0.0	0.0	0.0	0.001
Neurological disease (%)	0.0	0.0	1.0	1.6	0.0	0.0	0.0	NS

AI/ID, Autoimmune/autoinflammatory diseases; CID, Combined immunodeficiency; IBD, Inflammatory bowel disease; NS, not significant; PAD, Primary antibody deficiency.

## 4 Discussion

The present study provides a comprehensive overview of autoimmune and autoinflammatory manifestations in a large, nationally representative cohort of patients with IEI. Although autoimmunity and autoinflammation are a well-known features of IEI, the current literature, while providing valuable insights, does not capture the full panorama of these manifestations. There remains a clear need to define their spectrum in large, unselected series encompassing the full range of IEI categories, independent of patient age or disease subtype. The strength of such an approach lies in its ability to more precisely quantify the prevalence and distribution of autoimmunity and autoinflammation in IEI and to delineate patterns of clustering or segregation, thereby offering a true cartography of autoimmune and autoinflammatory manifestations in the context of these monogenic disorders. The implications for clinical practice are substantial, as they foster new approaches to patient evaluation and enhance clinician awareness of these complications. For instance, these findings underscore the importance of considering an autoimmune etiology in patients presenting with cytopenia in the context of hypomorphic RAG deficiency, and conversely, of evaluating for underlying immunodeficiency in patients with unexplained autoimmune cytopenia.

A total of 825 Algerian patients, originating from a highly consanguineous population and presenting with more than 80 distinct IEI across all 10 IEI categories, were enrolled in this study. Recruitment was nationwide and included both children and adults, without restriction regarding the type of IEI. Autoimmune and/or autoinflammatory manifestations were observed in 26.3% of patients, a prevalence comparable to that reported in a French study based on the French National PIDs Registry (26.2%) (12). Lower frequencies have been described in other cohorts, including Slovenian (22%) (14), Kuwaiti (20%) (15), and Iranian (20%) (16) patients. These inter-cohort differences may reflect variations in study design, patient age, disease duration, ethnic background, environmental exposures, cohort composition, and the criteria used to define autoimmune/autoinflammatory conditions. Taken together, these data indicate that autoimmune and autoinflammatory complications are common among patients with IEI, with a prevalence at least tenfold higher than in the general population (12).

A wide spectrum of autoimmune and autoinflammatory conditions was observed in our cohort. The most frequent were autoimmune cytopenias (11.4%), followed by gastrointestinal (7.8%), rheumatologic (5.3%), and endocrine (3.4%) disorders. These frequencies closely mirror those reported in the French cohort, which documented 12.3% for cytopenias, 9.5% for gastrointestinal disorders, and 5.0% and 3.2% for rheumatologic and endocrine disorders, respectively (12). Notably, autoimmune/autoinflammatory manifestations were often inaugural, representing the first clinical presentation in nearly half of affected patients and in 13% of the overall IEI cohort. Collectively, these findings highlight the importance of recognizing autoimmunity and autoinflammation as an early sentinel feature of underlying IEI and support the systematic immunological evaluation of any patient presenting with early-onset autoimmune cytopenia or IBD, even in the absence of overt infections.

A key finding of our study is that autoimmune and autoinflammatory manifestations constitute a common thread across the entire spectrum of IEI, encompassing T-cell, B-cell, and innate immune defects. This observation underscores that loss of immune tolerance and dysregulated inflammation may arise through multiple, distinct pathogenic mechanisms. Although virtually all IEI confer an increased susceptibility to these complications, the magnitude of risk varies between disease groups. Importantly, certain genetic defects emerge as ‘hotspots’ for autoimmunity (**Figure 2B**). Hypomorphic RAG deficiency illustrate the consequences of defective central tolerance, with autoimmune features present in 70% of patients with leaky SCID. In these patients, defective V(D)J recombination disrupts thymic crosstalk, leading to reduced Autoimmune Regulator (AIRE) expression and impaired presentation of tissue-restricted antigens (23). The resulting breakdown of negative selection allows autoreactive T cells to escape deletion, while the inflammatory milieu characteristic of RAG deficient patients further promotes expansion of autoreactive clones and compromises Tregs function (22).

CD3γ deficiency offers another illustrative example of how defect in central tolerance can drive autoimmunity. In our cohort, two patients were diagnosed with CD3γ deficiency, both presenting with recurrent infections alongside autoimmune cytopenias. Consistent with previously reported cases, they exhibited T-cell lymphopenia with markedly reduced surface TCRαβ/CD3 expression, leading to impaired TCR signaling. Rowe et al. demonstrated that the T-cell repertoire in CD3γ deficiency carries a molecular signature predisposing to autoimmunity, notably an enrichment of hydrophobic amino acids at positions 6 and 7 of the TRB-CDR3 region, a recognized biomarker of self-reactivity (24). Mechanistically, the strength of TCR signaling within the thymic medulla is crucial for shaping CD4^+^ T-cell fate: strong self-peptide recognition typically induces negative selection or diversion into the Tregs lineage. In the setting of CD3γ deficiency, impaired signaling perturbs this process, allowing autoreactive conventional T cells to evade deletion, expand in the periphery, and drive autoimmune pathology (25). In addition, patients with CD3γ deficiency exhibit a reduced proportion of Tregs, with restricted repertoire diversity and impaired suppressive function, thereby facilitating the autoimmune activity of escaping autoreactive clones (24).

Beyond defects in central tolerance, mutations affecting *FOXP3* and *LRBA* are consistently associated with autoimmune manifestations, emphasizing the critical role of Tregs and immune checkpoint in maintaining peripheral tolerance (26, 27). Recently, *IKZF2* variants have been linked to combined immunodeficiency with immune dysregulation, with only 13 cases reported to date, most harboring heterozygous variants (28–31). In our cohort, we identified two affected individuals from separate kindlers: one with a heterozygous variant and another with a homozygous one. The latter displayed a distinctive phenotype characterized by recurrent infections, craniofacial anomalies, hearing impairment, and immune dysregulation, including refractory Evans syndrome and autoimmune hepatitis. Helios is highly expressed in Tregs and is essential for preserving their suppressive, non-inflammatory function. It also acts as a checkpoint on effector T-cell activation, and its deficiency results in early hyperactivation followed by functional exhaustion, thereby predisposing patients to autoimmunity and immune dysregulation (29, 32).

Although complement deficiencies are generally associated with a low prevalence of autoimmune and autoinflammatory manifestations compared with other IEI groups, CD55 deficiency stands out for its high autoinflammatory burden. In our cohort, 4 of 8 patients exhibited autoinflammatory or autoimmune manifestations, with IBD-like mucosal inflammation documented in 3 (37.5%) patients. Traditionally linked to paroxysmal nocturnal hemoglobinuria, CD55 deficiency was recognized as a distinct entity when Ozen et al. identified homozygous loss-of-function mutations in CD55 as the cause of the ultra-rare autosomal recessive disorder (CHAPLE disease), characterized by complement hyperactivation, angiopathic thrombosis, and protein-losing enteropathy (33, 34). Beyond its hallmark features, CD55 deficiency confers an elevated risk of inflammatory colitis. The mechanisms underlying the intestinal tropism of inflammation remain incompletely understood. Evidence indicates that, unlike CD46 or CD59, CD55 is essential for maintaining intestinal lymphatic integrity and protecting lacteals from complement-mediated injury (35). Furthermore, CD55 functions as a costimulatory ligand for CD97 on CD4^+^ T cells, enhancing their activation, proliferation, and IL-10 production. In CD55-deficient patients, T cells exhibit heightened TNF-α production via C5aR1 signaling, coupled with reduced IL-10 secretion due to impaired CD97-mediated costimulation. These convergent immunological perturbations likely drive the pronounced susceptibility to IBD (35). Thus, CD55 deficiency represents a unique monogenic model in which uncontrolled complement activation perturbs intestinal immune homeostasis, ultimately promoting the initiation and progression of chronic inflammatory disease.

A primary objective of this study was to map the landscape of autoimmune and autoinflammatory manifestations across the spectrum of IEI. Our findings reveal striking, disease-specific patterns, with clear clustering of particular autoimmune manifestations within defined genetic defects (**Figure 4**). Autoimmune cytopenias were notably enriched in PIRDs such as ALPS, LRBA, and TPP2 deficiencies, and were also highly prevalent in T-cell disorders, including CD3γ deficiency and hypomorphic RAG deficiency. IBD clustered with RIPK1 deficiency and was prominent in actin-related IEI, including ARPC1B and DOCK8 deficiencies, as well as in CHAPLE disease and CGD. Endocrine autoimmunity, while a cardinal feature of APECED and IPEX syndrome, emerged as a prominent feature in patients with STAT1 gain-of-function mutations. Inflammatory granulomatous lung disease was a consistent feature of LRBA deficiency, whereas granulomatous inflammation, whether confined to the lungs or extending to other organs, was relatively common (9.9%) in CVID. Collectively, these observations demonstrate that autoimmune and autoinflammatory manifestations in IEI are not randomly distributed but follow recognizable clustering patterns, reflecting the underlying genetic and immunologic architecture of each disorder. They further suggest that distinct pathogenic mechanisms are driving different conditions, paving the way for more precise, targeted therapeutic strategies.

Despite these clinically meaningful findings, several limitations should be acknowledged. First, the retrospective design highlights the need for longitudinal follow-up to capture autoimmune complications that may arise later in the disease course. Second, the small sample sizes in certain IEI types-understandable given the ultra-rare nature of some entities-preclude definitive conclusions. Nevertheless, trends and patterns were observed, which warrant validation in larger international cohorts spanning multiple countries within the North African or MENA region. Third, our study included a limited number of patients with monogenic autoinflammatory disorders, which likely led to an underestimation of the prevalence of autoinflammatory manifestations in our series. Finally, the absence of a comparative analysis between patients with and without autoimmune/autoinflammatory manifestations within the same entities limits the ability to assess the contributions of other factors, including environmental triggers (e.g., infections), genetic modifiers, and additional influences.

## 5 Conclusion

Our findings highlight that autoimmune and autoinflammatory complications are not incidental but represent a recurrent theme across the IEI spectrum. While present in almost all categories, their burden and distribution vary, with certain defects acting as clear ‘hotspots’, such as IPEX syndrome, APECED, LRBA, RAG1, RAG2, CD3γ, Helios, ARPC1B, and CD55 deficiencies. These observations emphasize how defects in tolerance checkpoints or complement regulation can drive distinct autoimmune/inflammatory phenotypes. The recognition of clustering patterns, particularly autoimmune cytopenias, IBD, and endocrine autoimmunity, has direct clinical implications. These manifestations should be regarded as red flags, guiding targeted evaluation and prompting genetic screening in the appropriate context, for instance, hypomorphic RAG mutations in refractory cytopenia. Mapping such associations not only refines our understanding of pathogenesis but also provides a practical framework for earlier diagnosis and tailored management.

## Data Availability

The original contributions presented in the study are included in the article/**Supplementary Material**. Further inquiries can be directed to the corresponding author.

## Supplementary material

Supplementary Figure 1 Distribution of different autoimmune and autoinflammatory manifestations with respect to IEI categories.
