# Supplementary figures and images for: Mapping the landscape of autoimmunity and autoinflammation in inborn errors of immunity: broad distribution with distinct clustering patterns
Source: Front Immunol. 2025 Nov 28;16:1725282. doi: 10.3389/fimmu.2025.1725282 (PMC12698554; doi:10.3389/fimmu.2025.1725282)

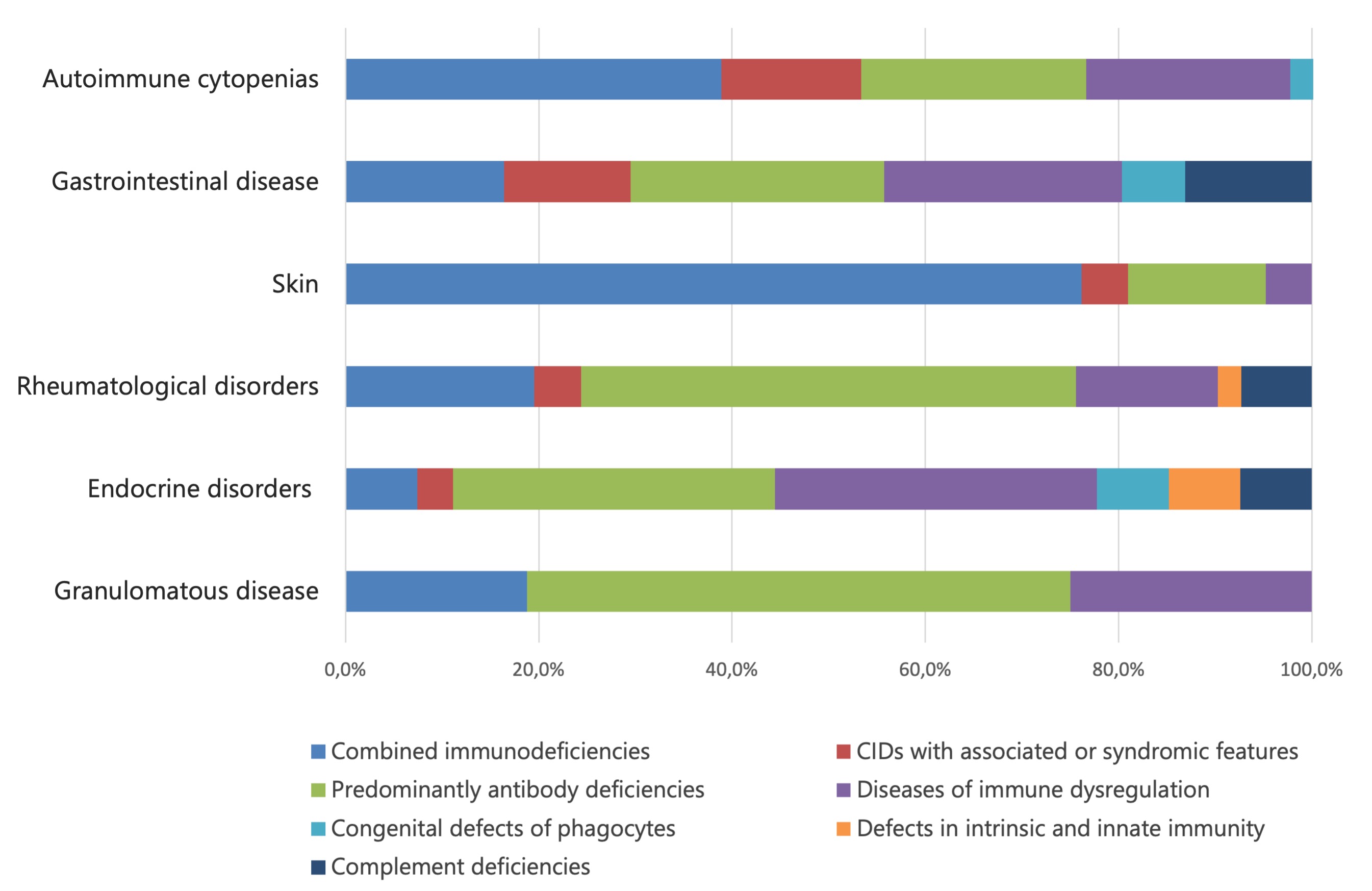

Supplement: Supplementary Figure 1 — Distribution of different autoimmune and autoinflammatory manifestations with respect to IEI categories. [file Image1.jpeg]
