# Supplementary material for: Mapping the landscape of autoimmunity and autoinflammation in inborn errors of immunity: broad distribution with distinct clustering patterns
Source: Front Immunol. 2025 Nov 28;16:1725282. doi: 10.3389/fimmu.2025.1725282 (PMC12698554; doi:10.3389/fimmu.2025.1725282)
Supplement: Supplementary file 2 [file Table1.docx]

**Supplementary Table S1:** Distribution of IEI in our cohort.

| **IEI type/category** | | **No.** | **Percent** |
| --- | --- | --- | --- |
| **CIDs** | | **208** | **25.2** |
| **SCIDs** | | 41 | 5.0 |
|  | T^–^B^–^NK^–^ SCID | 2 | 0.2 |
|  | T^–^B^–^NK^+^ SCID | 24 | 2.9 |
|  | T^–^B^+^NK^–^ SCID | 7 | 0.8 |
|  | T^–^B^+^NK^+^ SCID | 8 | 1.0 |
| **Omenn syndrome** | | 8 | 1.0 |
| **Leaky SCIDs** | | 19 | 2.3 |
|  | Hypomorphic RAG deficiency | 10 | 1.2 |
|  | Hypomorphic Artemis deficiency | 3 | 0.4 |
|  | Hypomorphic ADA deficiency | 2 | 0.2 |
|  | Hypomorphic Cernunnos deficiency | 1 | 0.1 |
|  | Hypomorphic DNA ligase IV deficiency | 1 | 0.1 |
|  | Hypomorphic Coronin 1A deficiency | 2 | 0.2 |
| ***Bona fide* CIDs** | | 140 | 17.0 |
|  | MHC class II deficiency | 43 | 5.2 |
|  | DOCK8 deficiency | 7 | 0.8 |
|  | CD40 deficiency | 2 | 0.2 |
|  | CD3γ deficiency | 2 | 0.2 |
|  | Helios deficiency | 2 | 0.2 |
|  | FCHO1 deficiency | 3 | 0.4 |
|  | MALT1 deficiency | 1 | 0.1 |
|  | IKBKB deficiency | 1 | 0.1 |
|  | SASH3 deficiency | 1 | 0.1 |
|  | GIMAP5 deficiency | 1 | 0.1 |
| **CIDs with associated or syndromic features** | | **131** | **15.9** |
|  | DiGeorge syndrome | 10 | 1.2 |
|  | Ataxia Telangiectasia | 16 | 1.9 |
|  | Wiskott-Aldrich syndrome | 20 | 2.4 |
|  | ARPC1B deficiency | 3 | 0.4 |
|  | Hyper-IgE syndrome | 74 | 9.0 |
| **Predominantly antibody deficiencies** | | **198** | **24.0** |
| Agammaglobulinemia | | 22 | 2.7 |
| Activated PI3K delta syndrome | | 4 | 0.5 |
| NFkB1 deficiency | | 1 | 0.1 |
| CVID | | 81 | 9.8 |
| Selective IgA deficiency | | 30 | 3.6 |
| **Diseases of immune dysregulation** | | **63** | **7.6** |
| APECED | | 2 | 0.2 |
| IPEX | | 1 | 0.1 |
| ALPS | | 5 | 0.6 |
| LRBA deficiency | | 3 | 0.4 |
| FERMT1 deficiency | | 1 | 0.1 |
| TPP2 deficiency | | 2 | 0.2 |
| RIPK1 deficiency | | 5 | 0.6 |
| CARMIL2 deficiency | | 2 | 0.2 |
| fHLH | | 5 | 0.6 |
| fHLH2 – PRF1 | | 1 | 0.1 |
| fHLH3 – UNC13D | | 1 | 0.1 |
| fHLH5 – STXBP2 | | 1 | 0.1 |
| Chediak-Higashi syndrome | | 10 | 1.2 |
| Griscelli syndrome type 2 | | 2 | 0.2 |
| **Congenital defects of phagocytes** | | **33** | **4.0** |
| Congenital neutropenia | | 15 | 1.8 |
| Chronic granulomatous disease | | 13 | 1.6 |
| LAD1 | | 4 | 0.5 |
| **Defects in intrinsic and innate immunity** | | **10** | **1.2** |
| MSMD | | 4 | 0.5 |
| STAT1 GOF | | 4 | 0.5 |
| **Autoinflammatory disorders** | | **3** | **0.4** |
| Blau syndrome | | 1 | 0.1 |
| Haploinsufficiency of A20 | | 1 | 0.1 |
| **Complement deficiencies** | | **155** | **18.8** |
| HAE | | 115 | 13.9 |
| C3 deficiency | | 2 | 0.2 |
| C7 deficiency | | 1 | 0.1 |
| Factor H deficiency | | 3 | 0.4 |
| Factor I deficiency | | 5 | 0.6 |
| CD46 deficiency | | 7 | 0.8 |
| CD55 deficiency (CHAPLE disease) | | 8 | 1.0 |
| **Bone marrow failure** | | **3** | **0.4** |
| Fanconi anemia | | 3 | 0.4 |
| **Phenocopies of IEIs** | | **3** | **0.4** |
| Good’s syndrome | | 3 | 0.4 |
| Unclassified immunodeficiencies | | **18** | **2.2** |

***Abbreviations:*** *ADA, Adenosine deaminase; ALPS, Autoimmune lymphoproliferative syndrome; APECED, Autoimmune polyendocrinopathy–candidiasis–ectodermal dystrophy; ARPC1B, Actin related protein 2/3 complex subunit 1B; CARMIL2, Capping protein regulator and myosin 1 linker 2; CHAPLE, Complement hyperactivation angiopathic thrombosis and protein-losing enteropathy; CID, Combined immunodeficiency; CVID, Common variable immunodeficiency; DOCK8, Dedicator of cytokinesis 8; FCHO1, F-BAR domain only protein 1; FERMT1, Fermitin family homolog 1; fHLH, Familial hemophagocytic lymphohistiocytosis; GOF, Gain-of-function; HAE, Hereditary angioedema; IEI, Inborn errors of immunity, IKBKB, Inhibitor of nuclear factor kappa-B kinase subunit beta; IPEX, Immune dysregulation–polyendocrinopathy–enteropathy–X-linked; LAD1, Leukocyte adhesion deficiency 1; LRBA, LPS-responsive beige-like anchor protein; MALT1, Mucosa-associated lymphoid tissue lymphoma translocation protein 1; MHC, Major histocompatibility complex; MSMD, Mendelian susceptibility to mycobacterial disease; NFkB1, Nuclear factor kappa B subunit 1; PRF1, Perforin; RAG, Recombination activating gene; RIPK1, Receptor-interacting serine/threonine-protein kinase 1; SASH3, SAM and SH3 domain-containing protein 3; SCID, Severe combined immunodeficiency; STXBP2, Syntaxin-binding protein 2; TPP2, Tripeptidyl peptidase II; UNC13D, Unc-13 homolog D.*
