# Supplementary material for: Mapping the landscape of autoimmunity and autoinflammation in inborn errors of immunity: broad distribution with distinct clustering patterns
Source: Front Immunol. 2025 Nov 28;16:1725282. doi: 10.3389/fimmu.2025.1725282 (PMC12698554; doi:10.3389/fimmu.2025.1725282)
Supplement: Supplementary file 3 [file Table2.docx]

**Supplementary Table S2:** Age at diagnosis and onset of symptoms and autoimmune/autoinflammatory manifestations by IEI category.

|  | **CIDs** | **CIDs with syndromic features** | **PADs** | **Diseases of immune dysregulation** | **Congenital defects of phagocytes** | **Defects in intrinsic and innate immunity** | **Complement deficiencies** | ***P*** |
| --- | --- | --- | --- | --- | --- | --- | --- | --- |
| Age at diagnosis,  median (IQR) | 19 (6 – 76) | 39 (14 – 72) | 96 (48 – 360) | 48 (17.2 – 129) | 30 (12 – 96) | 48 (28 – 90) | 288 (144 – 456) | **<0.0001** |
| Age at symptom onset, median (IQR) | 5 (1 – 18) | 5.5 (1 – 20) | 36 (6 – 168) | 12 (2 – 51) | 2.5 (1 – 24) | 7.5 (4.2 – 21) | 138 (72 – 243) | **<0.0001** |
| Age at onset of first^$^ autoimmune/autoinflammatory manifestation, median (IQR) | 36 (9 – 78) | 8 (2.5 – 64) | 114 (48 – 267) | 24 (3 – 90) | 179.5 (4.7 – 189) | 60 (30 – 99) | 294 (105 – 402) | **<0.0001** |

*^$^:* *Post-hoc pairwise comparisons were performed using Mann–Whitney U tests for all group pairs, with Bonferroni correction applied for multiple comparisons. With 7 groups, 21 pairwise comparisons were performed, resulting in a corrected significance threshold of α = 0.05/21 ≈ 0.00238. All observed pairwise comparisons listed below had raw P-values < 0.0001, which remained significant after Bonferroni correction. Significant differences were observed between the following group pairs:*

- *PADs*CIDs, PADs*CIDs with syndromic features, and PADs*Diseases of immune dysregulation.*
- *Complement deficiencies*CIDs, Complement deficiencies*CIDs with syndromic features, Complement deficiencies*Diseases of immune dysregulation, and Complement deficiencies*Defects of intrinsic and innate immunity.*

***Abbreviations:*** *CID, Combined immunodeficiency; IQR, interquartile range; PAD, Primary antibody deficiency.*
