# Supplementary material for: Mapping the landscape of autoimmunity and autoinflammation in inborn errors of immunity: broad distribution with distinct clustering patterns
Source: Front Immunol. 2025 Nov 28;16:1725282. doi: 10.3389/fimmu.2025.1725282 (PMC12698554; doi:10.3389/fimmu.2025.1725282)
Supplement: Supplementary file 4 [file Table3.docx]

**Supplementary Table S3:** Distribution of different autoimmune and autoinflammatory conditions across IEI categories.

|  | **No.** | **AI/ID**  (%) | **Autoimmune Cytopenia**  (%) | **Gastrointestinal disorders**  (%) | **Skin**  (%) | **Rheumatologic disorders**  (%) | **Endocrine disorders**  (%) | **Granulomatous disease**  (%) |
| --- | --- | --- | --- | --- | --- | --- | --- | --- |
| Combined immunodeficiencies | 208 | 30.8 | 16.8 | 4.8 | 7.7 | 3.8 | 1.0 | 1.4 |
| CIDs with associated or syndromic features | 131 | 15.3 | 9.9 | 6.1 | 0.8 | 1.5 | 0.8 | 0.0 |
| Predominantly antibody deficiencies | 198 | 31.3 | 10.6 | 8.1 | 1.5 | 10.6 | 4.5 | 4.5 |
| Diseases of immune dysregulation | 63 | 55.6 | 30.2 | 23,8 | 1.6 | 9.5 | 14.3 | 6.3 |
| Congenital defects of phagocytes | 33 | 24.2 | 9.1 | 12.1 | 0.0 | 0.0 | 6.1 | 0.0 |
| Defects in intrinsic and innate immunity | 10 | 40.0 | 0.0 | 0.0 | 0.0 | 10.0 | 20.0 | 0.0 |
| Complement deficiencies | 155 | 7.7 | 0.0 | 5.2 | 0.0 | 1.9 | 1.3 | 0.0 |

***Abbreviations:*** *AI/ID, Autoimmune/autoinflammatory disease; CID, combined immunodeficiency.*
